# Differences in Cerebral Perfusion Deficits in Mild Traumatic Brain Injury and Depression Using Single-Photon Emission Computed Tomography

**DOI:** 10.3389/fneur.2014.00158

**Published:** 2014-08-20

**Authors:** Kristoffer Romero, Sandra E. Black, Anthony Feinstein

**Affiliations:** ^1^Department of Psychiatry, Sunnybrook Health Sciences Centre, Toronto, ON, Canada; ^2^Heart and Stroke Foundation Centre for Stroke Recovery, Sunnybrook Health Sciences Centre, Toronto, ON, Canada; ^3^L. C. Campbell Cognitive Neurology Research Unit, Sunnybrook Health Sciences Centre, Toronto, ON, Canada

**Keywords:** TBI, depression, SPECT, neuropsychology, attention, partial least squares

## Abstract

**Background:** Numerous studies have shown decreased perfusion in the prefrontal cortex following mild traumatic brain injury (mTBI). However, similar hypoperfusion can also be observed in depression. Given the high prevalence of depressive symptoms following mTBI, it is unclear to what extent depression influences hypoperfusion in TBI.

**Methods:** Mild TBI patients without depressive symptoms (mTBI-noD, *n* = 39), TBI patients with depressive symptoms (mTBI-D, *n* = 13), and 15 patients with major depressive disorder (MDD), but no TBI were given 99m T-ECD single-photon emission computed tomography (SPECT) scans within 2 weeks of injury. All subjects completed tests of information processing speed, complex attention, and executive functioning, and a self-report questionnaire measuring symptoms of psychological distress. Between-group comparisons of quantified SPECT perfusion were undertaken using univariate and multivariate (partial least squares) analyses.

**Results:** mTBI-D and mTBI-noD groups did not differ in terms of cerebral perfusion. However, patients with MDD showed hypoperfusion compared to both TBI groups in several frontal (orbitofrontal, middle frontal, and superior frontal cortex), superior temporal, and posterior cingulate regions. The mTBI-D group showed poorer performance on a measure of complex attention and working memory compared to both the mTBI-noD and MDD groups.

**Conclusion:** These results suggest that depressive symptoms do not affect SPECT perfusion in the sub-acute phase following a mild TBI. Conversely, MDD is associated with hypoperfusion primarily in frontal regions.

## Introduction

Mild traumatic brain injury (mTBI) can be associated with cognitive dysfunction, post-concussive symptoms, and mood disturbances [for review, see Ref. (1)]. Cognitive deficits following mTBI usually occur on measures of attention, memory, and processing speed (2), with deficits typically resolving within the first 3 months of recovery. In terms of mood, depressive symptoms are the most frequent complaint, with approximately 26% of mTBI patients meeting the criteria for major depressive disorder (MDD) and an additional 20% reporting minor depressive symptoms 1-year post-injury (3, 4). Persisting cognitive deficits and disturbed mood are associated with poorer long-term outcome (4, 5).

Functional neuroimaging has consistently shown abnormalities in prefrontal regions in mTBI, which are associated with cognitive deficits. For example, functional magnetic resonance imaging (fMRI) activation is attenuated in mTBI patients when completing complex tasks such as the *n*-back, a measure of working memory (6, 7). Similarly, cerebral perfusion measured via single-photon emission computed tomography (SPECT) shows hypoperfusion in frontal regions that is associated with lower neuropsychological test scores (8–10). Studies measuring resting-state fMRI in mTBI also show decreased activity at rest following TBI compared to healthy controls (11, 12).

Notably, many of the regions affected by TBI are also associated with MDD without comorbid TBI. For example, MDD, in general, is also associated with frontal hypoperfusion on SPECT scans (13–16), decreased activation during cognitive task performance (17, 18), and alterations in resting-state activity (19, 20). Given these parallel findings among individuals with mTBI and MDD, it is unclear from a clinical standpoint whether decreased cerebral activity following mTBI may be attributed to neurological insult or depressive features. In addition, it is unclear whether patients with mTBI and concurrent depressive symptoms would show lower perfusion compared to patients with mTBI or MDD only.

Few studies have explored how depression may influence brain imaging in patients with mTBI, with most studies measuring structural differences among TBI patients with or without MDD (21–23). For example, Maller et al. (23) found decreased frontal white matter integrity in depressed TBI patients compared to non-depressed TBI patients, and Hudak et al. (22) found that the severity of depressive symptoms in a group of TBI patients correlated with atrophy in the orbitofrontal cortex. In one of the few studies examining the effect of depression on functional activation in TBI, Chen et al. (24) found that within a sample of concussed athletes, increasing severity of depressive symptoms was associated with attenuated prefrontal activation during a working memory task. However, to date, there have been no studies specifically examining how depressive symptoms post-TBI affect SPECT perfusion.

Given the uncertainty on how to interpret SPECT hypoperfusion in mTBI patients with concurrent depressive symptoms, and the fact SPECT remains an investigational tool for use in this patient population (25), we undertook a study with the aim of teasing apart the role of these competing variables on cerebral perfusion. Specifically, we compared cerebral perfusion between mTBI patients who did or did not endorse depressive symptoms on a general psychiatric screen and patients with MDD, using both quantitative measures of SPECT perfusion, as well as standard clinical ratings. We posited that group differences in perfusion would emerge primarily in medial prefrontal regions across all groups, with MDD patients exhibiting the lowest levels of perfusion, mTBI patients without depression showing the highest perfusion values, and mTBI patients with depressive symptoms exhibiting intermediary levels of perfusion.

## Materials and Methods

### Subjects

Fifty-two patients who sustained a mild TBI were recruited from the emergency room at Sunnybrook Health Sciences Centre (Table 1). Mild TBI was defined according to established criteria (26) as a score of 13 or greater on the Glasgow Coma Scale, loss of consciousness of <30 min, and a post-traumatic amnesia of 24 h or less. Diagnosis of mTBI was done through clinical interview by an experienced neuropsychiatrist. Exclusion criteria were the presence of a comorbid condition that could affect cognitive functioning (i.e., cerebrovascular disease), the presence of additional traumatic injuries or complications requiring hospital admission (e.g., fractures), current antidepressant use, and current substance abuse assessed by self-report or a urine screen for illicit substances.

**Table 1:** **Demographical and clinical information for TBI patients with depressive symptoms, TBI patients without depressive symptoms, and patients with MDD**.

	TBI-noD	TBI-D	MDD	*F-*test/*X*^2^	*p* Value
	*M* (SD)	*M* (SD)	*M* (SD)		
*n*	39	13	15	
Age	40.31 (10.15)	37.54 (10.20)	45.93 (8.55)	2.79	0.07
% Male	66.7	46.2	33.3	5.41	0.07
Estimated IQ (NART)	108.85 (11.09)	107.54 (11.81)	115.53 (8.09)	2.60	0.08
GCS				<1	0.835
13	3	1	–	
14	5	1	–	
15	28	11	–	
LOC				<1	0.472
None	0	0	–	
Altered consciousness	19	8	–		
<30 min	19	5	–		
PTA				1.53	0.464
None	9	1	–	
<1 h	21	8	–	
<24 h	9	4	–	
% Prior psychiatric diagnosis	7.70%	15.40%	–	<1	0.415
% Positive SPECT ratings	28.20%	46.20%	33.30%	1.43	0.49
Cognitive measures
Stroop (completion time, s)	24.86 (7.49)	30.65 (15.35)	28.37 (9.41)	1.95	0.15
SDMT (completion time, s)	101.75 (22.37)	117.72 (44.69)	108.78 (16.17)	1.78	0.18
**PVSAT (# errors)**	**7.97 (8.89)**	**18.08 (15.88)**	**9.00 (8.45)**	**4.61**	**<0.05**
Symptomatology measures
**GHQ-Somatic Subscale**	**4.05 (1.83)**	**6.00 (0.91)**	**3.20 (2.08)**	**9.34**	**<0.005**
**GHQ-Anxiety Subscale**	**2.05 (2.29)**	**5.00 (1.87)**	**3.47 (2.72)**	**8.35**	**<0.005**
**GHQ-Social Dysfunction Subscale**	**3.10 (2.41)**	**6.08 (1.70)**	**4.33 (2.99)**	**7.46**	**<0.005**
GHQ-Depression Subscale	0 (0)	1.69 (0.63)	4.33 (2.58)	–	–
**GHQ-Total Score**	**9.21 (5.50)**	**18.77 (3.52)**	**15.33 (9.39)**	**13.32**	**<0.005**

*mTBI-noD, TBI non-depressed group; mTBI-D, TBI depressed group; MDD, major depressive disorder group; NART, National Adult Reading Scale; GCS, Glasgow Coma Scale; LOC, loss of consciousness; PTA, post-traumatic amnesia; SPECT, single-photon emission computed tomography; SDMT, Symbol Digit Modalities Test; PVSAT, Paced Visual Serial Addition Test; GHQ, General Health Questionnaire*.

*Statistically significant effects are shown in bold*.

In addition, 15 patients with a diagnosis of MDD based on DSM-IV criteria were recruited from the outpatient psychiatry clinic at Sunnybrook. Patients with MDD were excluded if they had a previous history of TBI or other neurological insult.

### Clinical/cognitive assessment

Basic demographic information (age, sex, level of education) was obtained for each patient. Computerized versions of the Stroop test, symbol digit modalities test (SDMT), and paced visual serial addition test (PVSAT) were sent from a desktop computer to all patients (27). The Stroop test is a classic measure of response inhibition and executive functioning, requiring subjects to view a series of color names shown in congruent or incongruent colored ink and to respond by naming the color of the ink the word is shown in and not read the word. The SDMT is a measure of information processing speed and requires subjects to fill in a set of number–symbol pairings as quickly as possible. The PVSAT is a visual analog of the paced auditory serial addition test, a measure of complex attention and working memory. In this version, subjects saw a stream of single digit numbers and had to continuously report the sum of the last two numbers displayed. The National Adult Reading Test (NART) was given to estimate premorbid intellectual abilities (28).

Psychological distress was elicited with the 28-item General Health Questionnaire [GHQ; (29)]. This self-report questionnaire is used as a general screening tool of psychological distress and is scored on a 4-point scale and contains four subscales (anxiety, depression, social dysfunction, and somatic concern) of seven questions each with a choice of four responses per question. We used the method of scoring (0–0–1–1) advocated by the scale’s developer. Total scores, therefore, range from 0 to 28 with higher scores more indicative of psychopathology.

### Imaging acquisition

Single-photon emission computed tomography imaging was acquired within 2 weeks of neurological insult, using a triple-head gamma camera system (Prism 3000XP; Phillips Medical Systems Inc., Cleveland, OH, USA), 30–45 min after injection of 20 mCi (740 MBq) of Technetium-99m ethyl cysteinate dimer (^99m^Tc-ECD). Acquisition consisted of 120 slices obtained continuously over 360° (acquisition matrix = 128 × 128 pixels, zoom of 1.0), with an image resolution of 9.7 mm full-width half-maximum. Total acquisition time was 18.7 min. Reconstruction was performed using a ramp-filtered back projection algorithm with a 3D Wiener postfilter. Ellipses were fit to the approximate location on the outline of the head in each axial image, with an attenuation correction applied to each image, resulting in a voxel size of 2.17 mm × 2.17 mm × 3.56 mm. SPECT images were co-registered to an ROI SPECT template containing 75 ROIs using Automated Image Registration (AIR, version 3.0) (30). The template was created by co-registering a T1 MRI of a middle-aged male healthy control representative of the normal population, along the AC–PC axis to their SPECT scan, using a 12-parameter affine model and least squares fit with intensity scaling.

Finally, within each subject, for each ROI, a quantified metric of regional cerebral blood flow was calculated by measuring the mean perfusion count within the whole ROI and dividing it by the mean perfusion count within the cerebellum (31).

### Analysis

The 52 mTBI patients were classified into mTBI who reported any level of depressive symptoms (mTBI-D; *n* = 13) and mTBI who did not report any depressive symptoms (mTBI-noD; *n* = 39), based on the median of the depression subscale scores on the General Health Questionnaire, using the 0–0–1–1 scoring method outlined by the scale’s authors. The median depressive score was 0, therefore, we used a score of 1 or higher to differentiate between mTBI patients with depressive symptoms and mTBI patients without any depressive symptoms. Although the GHQ is used as a screening tool for psychological distress, factor analysis has confirmed that the depressive subscale is consistently found across several cultures (32). Demographical and clinical variables were compared between mTBI-D, mTBI-noD, and MDD groups with one-way ANOVAs or Chi-square statistics, using SPSS version 20.

### Imaging analysis

We compared quantified SPECT perfusion values across mTBI-D, mTBI-noD, and MDD groups using one-way ANOVAs. We focused on six regions bilaterally based on the established involvement of frontal and temporal hypoperfusion in both mild TBI and MDD. These regions (12 in total) included bilateral inferior frontal gyri, middle frontal gyri, superior frontal gyri, anterior cingulate gyri, orbitofrontal gyri, and hippocampi. We controlled multiple comparisons across the ROIs using a Bonferroni correction, with a significant *p* value set at 0.004. In addition, for any significant ROIs, Bonferroni-corrected tests of simple effects were conducted to compare SPECT perfusion across pairs of groups.

Single-photon emission computed tomography images were also analyzed using partial least squares [PLS; (33–35)]. PLS is a multivariate, data-driven technique that extracts latent variables that maximize the covariance between two matrices of data using singular value decomposition and is particularly suited for situations when the number of variables far exceeds the number of observations/subjects, and when many of those variables are highly correlated (i.e., different ROIs). Rather than the univariate method of running a large number of statistical tests on discrete regions or voxels, in PLS the entire group of ROIs is treated as a large matrix of data along with other data (in this case, vectors denoting group membership). These matrices are combined and decomposed via singular value decomposition to determine if there exists latent variable(s) that maximally explain the covariance between data matrices. For this study, we employed mean-centered PLS, which extracts patterns of SPECT perfusion across all ROIs, which maximally differentiate patient groups (i.e., how much variance in perfusion is accounted for by group membership). The analysis is similar to discriminant function analysis, such that in PLS the latent variables serve a similar role as discriminate functions. Several other studies have used PLS to differentiate between patient populations, using various imaging modalities (33). Using a data-driven technique such as PLS allows us to provide additional, hypothesis-free evidence to determine (a) whether patterns of brain perfusion can differentiate between different patient populations, (b) how different patient groups are differentiated, and (c) whether those brain regions include *a priori* ROIs. The significance of extracted latent variables is estimated using permutation testing, in which behavioral observations are shuffled within subjects, to create a distribution for comparison to the original extracted variable (i.e., whether the latent variable from the observed, experimental data is significantly different from a distribution of noise). For this study, 1000 permutations with a threshold of *p* < 0.05 were used. Because the statistical analysis is done in a single step, there is no need for correcting multiple comparisons across the whole brain. In addition, the reliability of each ROI’s contribution to the latent variables (i.e., salience) is calculated via bootstrap resampling, which estimates the standard error of the salience of each ROI’s contribution to the latent variable. Salience-to-standard error ratios derived through bootstrap resampling are approximately equivalent to *z*-scores and reflect the consistency of that brain region’s contribution to the extracted pattern. 500 bootstrap resamplings were used to construct the saliences; ROIs were considered reliable if the salience to standard error ratio exceeded 3.5 corresponding to a *p* value of 0.0005.

In addition, all SPECT scans were rated clinically as normal or abnormal by a radiologist or nuclear medicine specialist, who were both blind to group membership. All research was approved by the Research Ethics Board at Sunnybrook Health Sciences Center and all patients provided informed consent prior to participating.

## Results

There were no significant differences between groups in terms of age, sex, or estimated IQ (Table 1). In addition, mTBI-D and mTBI-noD groups did not differ on indices of TBI severity, i.e., GCS, LOC, duration of PTA, or the frequency of a premorbid psychiatric diagnosis.

In terms of cognitive performance, there was a significant overall difference between patient groups on the PVSAT, a measure of complex attention and working memory, *F*(2,63) = 4.61, *p* = 0.014: tests of simple effects confirm that mTBI-D patients showed more impaired performance (i.e., increased number of errors) than both the mTBI-noD patients (*p* < 0.005) and the MDD patients (*p* < 0.005) (Table 1).

Furthermore, there were no differences between groups in the percentage of patients with positive SPECT findings based on clinical ratings *F*(2,63) = 1.43, *p* = 0.49, suggesting that visual inspection by experienced radiologists is not sufficiently sensitive to capture any group differences in perfusion due to depressive symptoms (Table 1).

**Figure 1:**
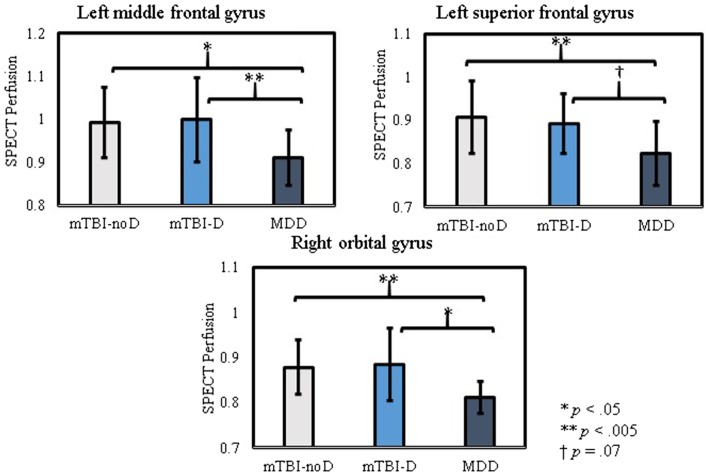
**Pairwise comparisons of SPECT perfusion in regions of interest with significantly different activity across patient groups**.

Conversely, one-way ANOVAs comparing quantified SPECT perfusion across groups showed significant differences in most of the frontal ROIs, with differences in the right orbitofrontal cortex, and left middle and superior frontal gyri surviving correction for multiple comparisons (Table 2). Pairwise comparison of perfusion revealed a similar pattern across these significant regions; the MDD group showed lower perfusion compared to both the mTBI-D or mTBI-noD groups and the mTBI groups did not significantly differ from each other (Figure 1).

**Table 2:** **Mean SPECT perfusion in regions of interest in TBI patients with or without depressive symptoms and patients with MDD**.

		SPECT perfusion			*F-*test	*p* Value
		mTBI-noD	mTBI-D	MDD	
		*M* (SD)	*M* (SD)	*M* (SD)	
L	Anterior cingulate gyrus	0.765 (0.084)	0.743 (0.098)	0.709 (0.092)	2.14	0.126
R	Anterior cingulate gyrus	0.774 (0.093)	0.764 (0.128)	0.734 (0.104)	<1	0.449
L	Inferior frontal gyrus	0.967 (0.081)	0.991 (0.084)	0.093 (0.086)	4.54	0.014
R	Inferior frontal gyrus	0.971 (0.084)	0.981 (0.114)	0.889 (0.077)	5.32	0.007
**L**	**Middle frontal gyrus**	**0.993 (0.082)**	**1.00 (0.098)**	**0.911 (0.065)**	**6.29**	**0.003**
R	Middle frontal gyrus	0.982 (0.086)	0.970 (0.105)	0.901 (0.072)	4.78	0.012
**L**	**Superior frontal gyrus**	**0.909 (0.084)**	**0.894 (0.069)**	**0.824 (0.073)**	**6.40**	**0.003**
R	Superior frontal gyrus	0.869 (0.086)	0.867 (0.060)	0.806 (0.073)	3.60	0.033
L	Orbital gyrus	0.801 (0.054)	0.819 (0.069)	0.750 (0.056)	5.85	0.005
**R**	**Orbital gyrus**	**0.878 (0.060)**	**0.885 (0.080)**	**0.811 (0.035)**	**7.58**	**0.001**
L	Hippocampus	0.639 (0.065)	0.635 (0.055)	0.657 (0.060)	<1	0.703
R	Hippocampus	0.614 (0.066)	0.614 (0.068)	0.605 (0.074)	<1	0.866

*mTBI-noD, mild traumatic brain injury patients without depressive symptoms*.

*mTBI-D, mild traumatic brain injury patients with depressive symptoms*.

*MDD, major depressive disorder patients*.

*Statistically significant effects are shown in bold*.

**Figure 2:**
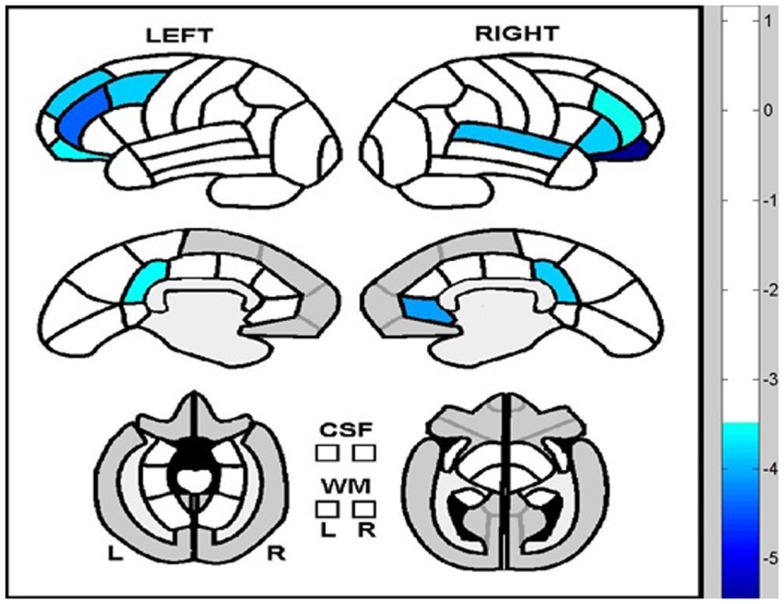
**Brain regions significantly differentiating depressed and non-depressed TBI patients from major depressive disorder patients**. Regions in blue show lower perfusion levels in depressed patients compared to both mTBI patient groups.

A similar picture emerged using a data-driven multivariate analysis. PLS analysis on SPECT perfusion across the whole brain yielded a pattern of perfusion that significantly dissociated the mTBI-D and mTBI-noD groups from the MDD group (*p* = 0.004). Crucially, the regions significantly contributing to this pattern had notable overlap with the *a priori* ROIs and included the bilateral orbitofrontal gyri, right inferior frontal gyrus, bilateral middle frontal gyri, left superior frontal gyrus, and right anterior cingulate cortex. In addition, other areas contributing to this pattern included the right superior temporal gyrus and bilateral posterior cingulate cortices (Table 3; Figure 2). For all these regions, perfusion was significantly lower in MDD patients compared to mTBI-noD and mTBI-D patients, bolstering the notion that depressive symptoms do not affect SPECT perfusion in the sub-acute phase following mTBI.

**Table 3:** **Brain regions comprising the pattern of SPECT perfusion that significantly differentiates TBI patient groups from the MDD group, using mean-centered partial least squares**.

	Brain Region	BSR
L	Orbitofrontal gyrus	−3.5472
R	Orbitofrontal gyrus	−5.467
R	Inferior frontal gyrus	−3.8382
L	Middle frontal gyrus	−4.3717
L	Middle frontal gyrus	−3.8253
R	Middle frontal gyrus	−3.5312
L	Superior frontal gyrus	−3.8617
R	Anterior cingulate cortex	−4.0715
L	Posterior cingulate cortex	−3.5965
R	Posterior cingulate cortex	−3.7877
R	Superior temporal gyrus	−3.9391

*BSR, bootstrap ratio; the ratio of salience to bootstrap-derived standard error is equivalent to a *z*-score of the contribution of each brain region to the latent variable differentiating mTBI-D and mTBI-noD groups from the MDD group*.

To determine whether SPECT perfusion was associated with individual differences in cognitive performance, we calculated the correlation between perfusion in the right orbitofrontal gyrus and PVSAT scores, within each patient group. However, there were no significant correlations between perfusion and PVSAT scores in any group (mTBI-D, *r* = 0.03, *p* = 0.88; mTBI-noD, Spearman’s ρ = −0.14, *p* = 0.65; MDD, Spearman’s ρ = 0.45, *p* = 0.1).

## Discussion

The most notable findings from this study were that quantified measures of SPECT perfusion failed to distinguish between mTBI subjects with and without depressive symptoms; however, significant hypoperfusion was found in subjects with a major depression but no TBI. These results were consistent using both univariate analysis of *a priori* ROIs and using a data-driven multivariate method. In addition, we found that mTBI patients with depressive symptoms showed worse performance on a measure of complex attention compared to both mTBI patients without depressive symptoms and MDD patients.

To our knowledge, this is the first investigation into the effect of depressive symptoms on SPECT perfusion in mTBI. Based on prior research showing both TBI and MDD subjects have prefrontal and temporal hypoperfusion (8, 16), we hypothesized that these regions would also be implicated in mTBI patients with depressive symptoms, however, SPECT perfusion did not differ between mTBI patients with or without depressive symptoms. Only a handful of studies in the TBI literature have examined cerebral correlates of depression, but the focus has been on structural measures (21–23). Maller et al. (23) compared diffusion tensor imaging (DTI) between TBI patients with MDD, TBI patients without MDD, patients with MDD but no TBI, and healthy controls, and found that TBI patients with MDD showed increased radial diffusivity (i.e., decreased white matter integrity) in the corpus callosum and dorsolateral prefrontal white matter compared with TBI patients without MDD. In addition, patients with MDD showed a similar pattern of increased diffusivity compared to healthy controls, suggesting that these findings are associated with MDD in general, independent of any TBI-related pathophysiological processes. Hudak et al. (22) examined individual differences in Beck Depression Inventory (BDI-II) scores within a group of TBI patients and found correlations with atrophy in the orbitofrontal cortex, suggesting that gray matter loss in this region following mTBI may be partially attributed to depression. Other studies examining depressive symptoms and neuroimaging in TBI have similarly examined patients in the chronic phase (24). Thus, these findings cannot be directly compared with our study, as we obtained SPECT images in the sub-acute phase rather than several months post-injury. One possibility is that within the TBI patient population, neuroimaging may only be sensitive to persisting depressive symptoms and not the presence psychiatric complaints in the more acute phases.

Similarly, it should be noted that the endorsement of depressive symptoms is not equivalent to a diagnosis of MDD, both in terms of symptom severity and duration. It may be the case that reporting depressive symptoms in the sub-acute phase following a TBI is tied more closely to the presence of post-concussive symptoms (i.e., somatic, cognitive complaints), factors that may differ from the factors contributing to MDD in those without cerebral trauma. Our data are consistent with this notion in that there were no significant differences in SPECT perfusion among MDD patients and either mTBI groups, perhaps suggesting separate etiologies and neural substrates associated with depressive symptoms in these groups.

Our finding of decreased cerebral perfusion in the MDD group is consistent with data in the general psychiatry literature showing that MDD is associated with cerebral abnormalities in prefrontal and temporal regions, specifically, orbitofrontal cortex, anterior cingulate cortex, middle and superior frontal regions, as well as the hippocampus and amygdala (17–19, 36). In terms of SPECT, decreased perfusion is typically observed in prefrontal regions, with perfusion levels correlating with symptom severity (13, 37) [but see Ref. (20)]. Current theories of depression implicate limbic and prefrontal regions in a dynamic interaction between altered resting-levels of neural activity and heightened reactivity to emotional stimuli, corresponding to the ruminative tendencies and negative biases commonly observed in these patients (19, 20). Although existing studies have only compared SPECT perfusion between MDD patients and healthy controls, given that both TBI and MDD are associated with frontal hypoperfusion, our data indicate that the magnitude of frontal hypoperfusion in MDD is relatively greater than the decrease in perfusion observed following mTBI.

Taken together, the present findings suggest that mild levels of depressive symptoms following mTBI do not contribute to sub-acute cerebral hypoperfusion, as quantified measures of SPECT perfusion were unable to distinguish among mTBI patients with or without depressive symptoms. Thus, it seems that in patients with mTBI, SPECT is not sufficiently sensitive to differentiate the presence or absence of milder levels of depressive symptoms. Whether SPECT is able to distinguish between mTBI patients with or without major depression is not clear; extrapolating from our present findings and given the ability of other imaging modalities to distinguish TBI patients with MDD from those without MDD, we posit that quantified metrics of SPECT perfusion may indeed be able to make such distinctions.

Although SPECT remains an investigational tool for this patient population (25), the results from the present study suggest that it may have clinical utility that warrants further exploration. Indeed, a recent systematic review found that SPECT is potentially more sensitive to cerebral dysfunction not always evident on non-contrast CT or MRI. Moreover, SPECT was found to have almost 100% negative predictive ability (38). It may be that SPECT or other measures of cerebral perfusion or resting-state neural activity could be more sensitive to cerebral dysfunction compared to CT or MRI in the more acute/sub-acute phases, when morphological differences likely would not yet have fully emerged.

In contrast, SPECT perfusion measured by *clinical* ratings was not able to differentiate any patient groups, suggesting that the differences in perfusion may only emerge using quantified metrics and multivariate analysis rather than visual ratings. Multivariate approaches to analyzing brain imaging are becoming more common, as our understanding of the complex networks underlying psychopathology continues to develop (36, 39, 40). Advantages of PLS are that it is a data-driven method with few *a priori* assumptions and the resultant patterns of brain activity are constrained to the topic of interest, in this case, maximally differentiating patient groups. Other multivariate methods such as independent components analysis also have few prior assumptions but require some subjective selection with respect to which networks to analyze further (41).

An additional finding was that TBI patients with depressive symptoms showed poorer cognitive performance compared to both TBI patients without depressive symptoms and MDD patients. This is consistent with evidence showing that the presence of mood disturbances in TBI is associated with cognitive dysfunction (42), poorer long-term outcome (4), and gray matter atrophy (43). However, the group differences in cognitive performance did not mirror group differences in SPECT and SPECT perfusion was not correlated with PVSAT scores, suggesting that it is not sensitive to changes in cognitive performance associated with disturbed mood.

A limitation to the present investigation was the relatively small sample size. In addition, although the GHQ is a widely used screening tool for psychological distress, it does not provide a more in-depth assessment of depressive symptomatology. Future studies should compare SPECT perfusion in TBI patients with and without a formal diagnosis of MDD. Furthermore, we did not have a healthy control sample with which to compare perfusion from the different patient populations. Given that most existing research on TBI patients and SPECT used clinical ratings of perfusion, there are no existing studies using equivalent methods to provide a normal range of normal perfusion values in a healthy population. Also, although there were no significant differences between groups in terms of age or IQ, there was a numerical trend toward increasing age and IQ in the MDD group. Subsequent studies should utilize larger samples and include more stringently matched groups along with healthy controls, as well as collect more comprehensive measures of cognitive functioning and symptomatology.

In sum, the present findings are of direct clinical relevance for two reasons: (1) both TBI in general and depression following TBI are common, and (2) SPECT is a frequently used tool to investigate cerebral abnormalities (44, 45). We found that SPECT perfusion can differentiate among patients with MDD and mTBI but only through using quantified measures of perfusion and not standard clinical ratings. However, SPECT could not differentiate among mTBI patients with or without depressive symptoms, suggesting that it may only be useful with patients whose symptoms reach the severity of a MDD.

## Author Contributions

Kristoffer Romero helped in analysis and interpretation of the data, as well as preparation and revision of the manuscript. Sandra E. Black and Anthony Feinstein aided in data acquisition, as well as in preparation and revision of the manuscript.

## Conflict of Interest Statement

Dr. Kristoffer Romero reported no biomedical financial interests or potential conflicts of interest. Dr. Sandra E. Black is an *ad hoc* consultant for General Electric. Dr. Anthony Feinstein was funded by the Canadian Institute of Health Research (grant # 36535).
